# The representation of protein complexes in the Protein Ontology (PRO)

**DOI:** 10.1186/1471-2105-12-371

**Published:** 2011-09-19

**Authors:** Carol J Bult, Harold J Drabkin, Alexei Evsikov, Darren Natale, Cecilia Arighi, Natalia Roberts, Alan Ruttenberg, Peter D'Eustachio, Barry Smith, Judith A Blake, Cathy Wu

**Affiliations:** 1 The Jackson Laboratory, 600 Main St., Bar Harbor, ME, 04609, USA; 2 Protein Information Resource, Dept of Biochemistry and Molecular & Cellular Biology, Georgetown University Medical Center, 3300 Whitehaven St., NW, Washington, DC, 20007, USA; 3 Center for Bioinformatics and Computational Biology, University of Delaware, 15 Innovation Way, Suite 205, Newark, DE 19711, USA; 4 School of Dental Medicine, State University of New York at Buffalo, 349 Squire Hall, Buffalo, NY, 14214, USA; 5 Department of Biochemistry, NYU School of Medicine, 550 First Avenue, New York, NY 10016, USA; 6 Department of Philosophy and Center of Excellence in Bioinformatics and Life Sciences, State University of New York at Buffalo, 130 Park Hall, Amherst, NY, 12460, USA

## Abstract

**Background:**

Representing species-specific proteins and protein complexes in ontologies that are both human- and machine-readable facilitates the retrieval, analysis, and interpretation of genome-scale data sets. Although existing protin-centric informatics resources provide the biomedical research community with well-curated compendia of protein sequence and structure, these resources lack formal ontological representations of the relationships among the proteins themselves. The Protein Ontology (PRO) Consortium is filling this informatics resource gap by developing ontological representations and relationships among proteins and their variants and modified forms. Because proteins are often functional only as members of stable protein complexes, the PRO Consortium, in collaboration with existing protein and pathway databases, has launched a new initiative to implement logical and consistent representation of protein complexes.

**Description:**

We describe here how the PRO Consortium is meeting the challenge of representing species-specific protein complexes, how protein complex representation in PRO supports annotation of protein complexes and comparative biology, and how PRO is being integrated into existing community bioinformatics resources. The PRO resource is accessible at http://pir.georgetown.edu/pro/.

**Conclusion:**

PRO is a unique database resource for species-specific protein complexes. PRO facilitates robust annotation of variations in composition and function contexts for protein complexes within and between species.

## Background

Logical and semantic access to related protein forms is critical for advancing bioinformatics approaches to representing, modeling, and reasoning about complex biological systems at the genomic and cellular level [1]. The Protein Ontology (PRO) Consortium develops and maintains ontology resources for the representation of protein forms for all organisms [2]. PRO is one of the six inaugural ontologies that form the Open Biological and Biomedical Ontologies (OBO) Foundry [3].

The PRO has three components informally referred to as ProForm, ProEvo, and ProComp [4]. ProForm represents species-specific and species-independent classes of protein isoforms, co- and post-translationally modified forms, and variant forms. ProEvo represents evolutionary relatedness of proteins. ProComp, the focus of this manuscript, represents multi-protein complexes, with an initial (but not exclusive) emphasis on protein components of complexes in mouse and human. ProComp, uses the Gene Ontology [5] (GO) definition of protein complex: "Any macromolecular complex composed of two or more polypeptide subunits, which may or may not be identical. Protein complexes may have other associated non-protein prosthetic groups, such as nucleotides, metal ions or other small molecules." (GO:0043234). Therefore protein complexes may, in addition, have components that are nucleic acids, carbohydrates, or lipids. Protein complexes are distinguished from protein-protein *interactions *in that they are continuant entities, i.e. they endure or continue to exist through time. Interactions, in contrast, are occurrent entities, i.e. they occur in time through successive temporal phases. The explicit representation of protein complexes in PRO--defining each member of the complex at the level of its isoform, variant, or modified form--provides the ability to represent complex biological knowledge as it is emerging in the experimental research community in structures that are both human readable and accessible to algorithmic approaches.

**Figure 1:**
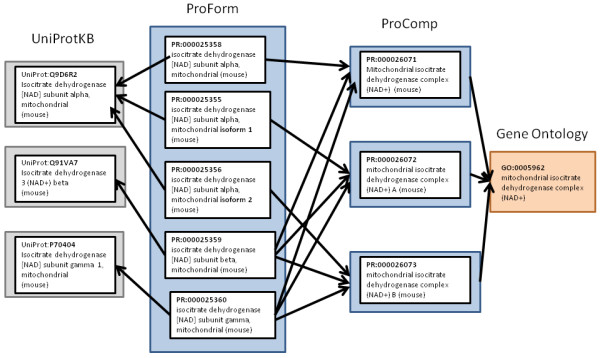
**Schematic showing the relationships between UniProtKB, ProForm, GO, and ProComp**. Arrows between ProForm and UniProtKB are *xref*, those between ProComp and ProForm are *has_part*, and between ProComp and Gene Ontology the arrows depict *is_a *relationships. In ProForm each protein form is assigned a unique identifier and is cross-referenced to protein entries in UniProtKB. Protein isoforms and modified forms are described in UniProtKB records, but in contrast to PRO, each protein form is not represented as a separate, uniquely accessioned entity in UniProtKB. For example, for the alpha subunit of IDH in mouse there is a PRO entry for the protein (PR:000025358) and for each of the alpha protein isoforms (PR:000025355 and PR:000025356). In UniProt the IDH alpha subunit and its isoforms are all represented in the same record (UniProt: Q9D6R2). In ProComp, accessioned, species-specific protein complex entities are described using protein entries from ProForm. The protein complexes in ProComp are cross-referenced to species-independent complex representations in the Gene Ontology (GO).

ProComp leverages, and cross references, entries in existing protein-centric informatics resources, including the protein complexes that are represented in the Cellular Component branch of the Gene Ontology. In the GO, types of protein complexes are defined in terms of constituent macromolecule classes and the function(s) that the complexes carry out. By agreement within the protein informatics community, PRO represents the species-*specific *classes of protein complexes, while GO, in most instances, represents the species-*independent *classes of protein complexes; within PRO, the latter are referred to by using GO identifiers. The UniProt Knowledgebase (UniProtKB) [6,7] describes protein complexes, though not as separate accessioned entities. References to protein records in UniProtKB are made through entries in the ProForm sub-ontology within PRO (Figure 1). A major contribution of PRO as a protein biology community informatics resource is that it provides a formal ontological structure with foundation in Basic Formal Ontology http://www.ifomis.org/bfo/to describe types of protein complexes and gives these types unique, permanent identifiers http://www.obofoundry.org/id-policy.shtml. PRO facilitates functional annotation of proteins and protein complexes with specific cellular contexts; it promotes compatibility with other ontology resources; and it promotes use of software tools for reasoning and analysis.

Uniquely among protein biology community informatics resources, PRO follows a principles-based strategy for ensuring ontological adequacy (i.e. biologically accurate, logically coherent and computationally useful). Some of these principles flow from the PRO's membership of the OBO (Open Biological and Biomedical Ontologies) Foundry. The OBO Foundry requires the use of Basic Formal Ontology http://www.ifomis.org/bfo/ as the top-level formal ontological structure; this ensures compliance with standard methodologies for ensuring formal adequacy such as OntoClean [8]. The OBO Foundry also requires the use of a standard formal syntax (we maintain versions in both OWL and OBO, thus allowing not only use of logical reasoners for consistency checking, but also providing an added layer of quality assurance by requiring the two versions to satisfy the formal principles needed for the two-way OBO-to-OWL mapping tools to run effectively). In addition, the OBO Foundry requires that an ontology is maintained and regularly updated to keep pace with scientific advances, rectify errors, and fill gaps identified by its users. Other principles to ensure ontological adequacy apply specifically to the PRO and to its constituent branches, including ProComp.

The development of ProComp, in particular, relies on high-level scientific content deriving from research on cellular contexts, diseases and, model organisms in addition to protein biology and chemistry. This requirement is addressed by a final OBO Foundry principle, which requires that each ontology is maintained in such a way as to involve the developers and users of other neighboring ontologies (e.g., GO, ProForm, ProEvo, etc.) in order to ensure consistency of scientific content. The ongoing process of critical review by representatives of multiple complementary disciplines is designed to guarantee that the ontology is developed on the basis of the best current scientific understanding of relevant subject-matters in each of these disciplines.

In this communication we present elements of PRO's current approach for representing species-specific types of protein complex, illustrate how they are being integrated with existing pathway database resources, and describe how the ProComp effort facilitates comparative protein biology and functional genomics.

### Construction and Content

#### Representation of protein complexes

The Protein Ontology is authored using the OBO 1.2 format [9]. The OBO format supports algorithmic reasoning and is also human readable. Terms in an OBO ontology are described by "stanzas." Each OBO stanza has an identifier, term name, and a textual term definition; the stanzas may also contain comments and synonyms. A series of relationship declarations are used to construct a logical definition. The declarations relate the term to other terms within the PRO ontology as well as to other ontologies, such as the GO (Figure 1).

The protein complex types in PRO are defined by means of multiple part relationships to PRO-defined species-specific protein types. The species-specific protein types can be of isoforms or modified isoforms. Each PRO complex stanza includes an *is_a *(i.e. subtype) relationship to the corresponding Gene Ontology (GO) protein complex term (Figure 1). Cross references to protein entries in UniProtKB are not included in protein complex stanzas. Instead they are part of the stanzas that represent the component proteins in ProForm (Figure 1).

The initial development of ProComp makes use of examples of known protein complexes drawn from well-established, manually-curated pathway databases: EcoCyc [10], MouseCyc [11] and Reactome [12,13]. The close collaboration of PRO with these databases ensures that PRO allocates effort where there is demonstrated need, and that PRO's definitions are accessible to the biomedical research community in the functional context of cellular pathways. EcoCyc http://ecocyc.org represents transcriptional regulation, transporters, and biochemical pathways for *Escherichia coli*. MouseCyc http://mousecyc.jax.org focuses on pathways involved in biosynthesis, degradation, energy production, and detoxification. MouseCyc is unique among mammalian pathway resources in that the database is connected with the rich biological knowledge about mouse gene function and phenotypes contained in the Mouse Genome Informatics database (MGI; http://www.informatics.jax.org) [14]. Reactome is a manually curated knowledgebase of human biological pathways http://www.reactome.org. In Reactome, pathways are represented as series of molecular events that transform one or more input physical entities into one or more output entities, catalyzed or regulated by other entities. Entities include small molecules, proteins, post-translationally modified proteins, and complexes. Current priorities for ProComp are to create entries for all protein complexes in the contributing databases with an emphasis first on those with published species-specific experimental evidence and those with human disease relevance. Although ProComp is being populated initially from a few targeted database resources, information about protein complexes will also be obtained from other database resources such as IntAct [15] and the primary published literature.

### Utility and Discussion

Below we describe four use cases to highlight key elements in the representation of protein complexes in PRO and to illustrate how aspects of biological knowledge and complexity are being captured in ProComp.

#### Use Case 1. The 3-methylcrotonyl carboxylase (MCC) Complex: A heterodimeric complex

The MCC protein complex (E.C. 6.4.1.4) is a mitochondrial, biotin-dependent heterodimeric enzyme consisting of alpha and beta subunits. The PRO stanza for the murine MCC complex is illustrated below with key points illustrated by bold text. The ID for this protein complex type (PR:000025760 = http://purl.obolibrary.org/obo/PR_000025760) is unique, permanent, and is resolvable on the web to useful information. This class of complex is a subclass of the class of species-nonspecific MCC protein complexes defined in the Gene Ontology (GO:0002169) and this relationship is explicitly represented using an *is_a *statement in the stanza. The species specificity of the protein complex is indicated by the value of the *only_in_taxon *tag. The MCC complex has two components, an alpha and beta subunit represented by the *has_part *relationships that assert that each instance of the complex contains an instance of indicated peptide. The embedded cardinality declaration indicates the number of instances of that type of subunit in the complex. In the case of murine MCC, the cardinality declarations indicate that each complex has exactly one of each subunit (alpha-beta). The absence of a cardinality declaration indicates there is at least one copy of each subunit.

We deliberately use *has_part *to relate the complex to the subunit rather than *part_of *to relate the subunit to the complex. The latter protein-centric statement would indicate that each instance of the peptide is part of some instances of the indicated complex, which is not necessarily the case, as the peptide could exist in a free form or as part of other complexes. Finally, that this is a protein complex only found in mouse is represented by tag *only_in_taxon *paired with the official taxon identifier. The totality of these relationship statements makes up the logical definition for this complex type.

[Term]

id: PR:000025760

name: methylcrotonoyl-CoA carboxylase complex, mitochondrial (mouse)

def: "A methylcrotonyl-CoA carboxylase complex, mitochondrial, whose components are encoded in the genome of mouse." [PRO:CJB]

comment: Category = organism-complex. Entities of this type are disposed to have the enzymatic activity described by EC:6.4.1.4.

synonym: "beta-methylcrotonyl-CoA carboxylase (mouse)" EXACT []

synonym: "MCC (mouse)" EXACT []

is_a: GO:0002169! 3-methylcrotonyl-CoA carboxylase complex, mitochondrial

relationship: *has_part *PR:000025354 **{cardinality = "1"**} ! methylcrotonoyl-CoA carboxylase subunit alpha, mitochondrial (mouse)

relationship: *has_part *PR:000025357 **{cardinality = "1"} **! methylcrotonoyl-CoA carboxylase beta chain, mitochondrial (mouse)

relationship: only_in_taxon taxon:10090 ! Mus musculus

#### Use Case 2. The serine palmitoyltransferase (SPT) Complex: Differences in prokaryotic and eukaryotic protein complex organization

Serine palmitoyltransferase (SPT; EC 2.3.1.50) catalyzes the key reaction in the biosynthesis of sphingolipids. In many eukaryotic species, this enzyme is a heterodimer consisting of two subunits, SPTLC1 and SPTLC2. Recently, it was shown that in human cells, SPTLC1 and SPTLC2 form a complex with a third subunit, SPTLC3, with a resulting molecular mass of 480 kDa [16]. SPTLC1 is a common subunit to two core complexes SPTLC1-SPTLC2 and SPTLC1- SPTLC3, and it is likely that the ratio of SPTLC2 to SPTLC3 subunits in the SPT complexes, as well as binding of some smaller regulatory subunits, confers preferential activity of SPT complexes to specific acyl-CoA substrates [17,18].

**Figure 2:**
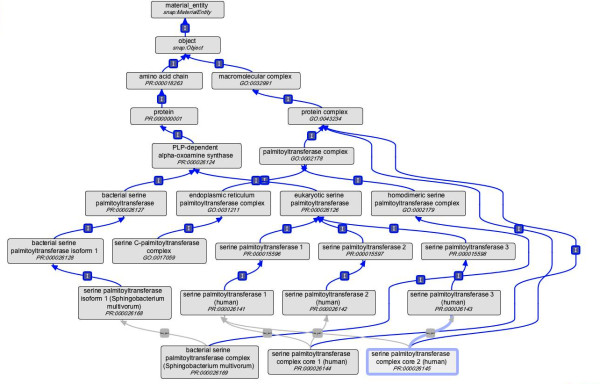
**PRO hierarchy depicting relationship for the human and bacterial SPT complex protein subunits, and for the SPT complexes**. The blue arrows and "I" icons represent *is_a *relationships among the entities. The protein components have PR ids and the complex concepts have Gene Ontology (GO) ids.

A serine palmitoyltransferase complex is also found in gram-negative sphingolipid-containing bacteria. Unusually, the outer membranes of these bacteria contain glycosphingolipid (GSL) instead of lipopolysaccharide, and SPT catalyzes the first step of the GSL biosynthetic pathway in these organisms. But, as opposed to the human SPT complex, bacterial SPT complex is homodimeric [19-21]. In some bacterial species the complex is water-soluble, whereas in eukaryotes the complex is membrane-bound. The component subunits of bacterial and eukaryotic SPT complexes are evolutionarily-related, and this relationship is evidenced in the ontology (Figure 2; both the bacterial and eukaryotic serine palmitoyltransferases trace back to the same progenitor class of pyridoxal 5'-phosphate (PLP)-dependent alpha-oxoamine synthase).

The representation of SPT complexes in PRO shown below and illustrated in Figure 2 highlights the differences in composition among human complexes and between the human and bacterial complexes. Stanzas for two human SPT complexes are depicted below. The core complex is represented by PR:000026144 while PR:000026145 represents the multimeric functional complex. The complex composition is formalized using the *has_part *relation to the corresponding classes of protein components. The functional human complex classes have an *is_a *relationship to the serine C-palmitoyltransferase complex class in GO (GO:0017059). The complex core has an *is_a *relationship to GO:0043234 (protein complex) as there is currently not a more specific complex class in GO.

[Term]

id: PR:000026144

name: serine palmitoyltransferase complex core 1 (human)

def: "A serine palmitoyltransferase complex that is heterodimeric consisting of one subunit of serine palmitoyltransferase 1 and serine palmitoyltransferase 2. These components are encoded in the genome of human." [PRO:CNA]

is_a: GO:0043234 ! protein complex

relationship: has_part PR:000026141 {cardinality = "1"} ! serine palmitoyltransferase 1 (human)

relationship: has_part PR:000026142 {cardinality = "1"} ! serine palmitoyltransferase 2 (human)

relationship: only_in_taxon taxon:9606 ! Homo sapiens

[Term]

id: PR:000026155

name: serine palmitoyltransferase complex A (human)

def: "A serine palmitoyltransferase complex consisting of an unknown combination of the serine

palmitoyltransferase subunits 1-3. The stoichiometry is a tetramer composed of the serine

palmitoyltransferase core complexes 1 and/or 2." [PMID:17331073, PRO:CNA]

comment: Category = organism-complex. Entities of this type are disposed to have the enzymatic activity described by EC:2.3.1.50.

is_a: GO:0017059 ! serine C-palmitoyltransferase complex

relationship: has_part PR:000026141 {cardinality = "4"}! serine palmitoyltransferase 1 (human)

relationship: has_part PR:000026142 ! serine palmitoyltransferase 2 (human)

relationship: has_part PR:000026143 ! serine palmitoyltransferase 3 (human)

relationship: only_in_taxon taxon:9606 ! Homo sapiens

The protein complex stanza for the bacterial SPT complex (PR:000026169) is shown below. The bacterial and human complexes differ in composition (Figure 2) but perform closely similar biochemical functions.

[Term]

id: PR:000026169

name: bacterial serine palmitoyltransferase complex (Sphingobacterium multivorum)

def: "A homodimeric serine palmitoyltransferase complex that is composed of bacterial serine palmitoyltransferase encoded in the genome of Sphingobacterium multivorum." [PRO:CNA, PMID:17557831, PMID:19564159]

comment: Category = organism-complex. Entities of this type are disposed to have the enzymatic activity described by EC:2.3.1.50.

is_a: GO:0002179 ! homodimeric serine palmitoyltransferase complex

relationship: has_part PR:000026168 {cardinality = "2"} ! bacterial serine palmitoyltransferase isoform 1 (Sphingobacterium multivorum)

relationship: only_in_taxon taxon:28454 ! Sphingobacterium multivorum

#### Use Case 3: Mitochondrial Isocitrate Dehydrogenase Complex; A heterotrimeric complex using different isoforms for one subunit

There are three types of isocitrate dehydrogenases (IDHs) in mammals; two IDHs utilize NADP^+ ^as a cofactor in the oxidative decarboxylation of isocitrate and one utilizes NAD^+^. The NAD^+^-dependent IDH is a mitochondrial protein complex in the citric acid cycle; it consists of three subunits (alpha, beta, gamma), each of which is encoded by a single gene. The mouse alpha subunit gene encodes two different protein isoforms by alternative splicing. ProComp can distinguish between complexes that differ in the isoform of one of the constituents. As illustrated below (in bold), the protein complex stanzas from PRO describe two types of IDH complexes that differ only in the type of isoform of the alpha subunit. Both classes of isoform-specific protein complexes shown below (PR:000026072 and PR:000026073) are subclasses of the mouse-specific protein complex class (PR:000026071) which, in turn, is a subclass of the species-independent complex class defined in GO (GO:0005962).

[Term]

id: PR:000026071

name: mitochondrial isocitrate dehydrogenase complex (NAD+) (mouse)

definition: A mitochondrial isocitrate dehydrogenase complex using NAD+ whose components are encoded in the genome of mouse [PRO:hjd]

is_a:GO:0005962 ! mitochondrial isocitrate dehydrogenase complex (NAD+)

relationship: has_part PR:000025358 ! isocitrate dehydrogenase [NAD] subunit alpha, mitochondrial (mouse)

relationship: has_part PR:000025359 ! isocitrate dehydrogenase [NAD] subunit beta, mitochondrial (mouse)

relationship: has_part PR:000025360 ! isocitrate dehydrogenase [NAD] subunit gamma, mitochondrial (mouse)

relationship: only_in_taxon taxon:10090! Mus musculus

[Term]

id: PR:000026072

name: mitochondrial isocitrate dehydrogenase complex (NAD+) A (mouse)

def: A mitochondrial isocitrate dehydrogenase complex using NAD+ whose components are encoded in the genome of mouse containing isoform 1 of subunit alpha" [PRO:hjd]

is_a:PR: 000026071! mitochondrial isocitrate dehydrogenase complex (NAD+) (mouse)

relationship: has_part PR:000025355 ! isocitrate dehydrogenase [NAD] subunit alpha, mitochondrial isoform 1 (mouse)

relationship: has_part PR:000025359 ! isocitrate dehydrogenase [NAD] subunit beta, mitochondrial (mouse)

relationship: has_part PR:000025360 ! isocitrate dehydrogenase [NAD] subunit gamma, mitochondrial (mouse)

relationship: only_in_taxon taxon:10090 ! Mus musculus

[Term]

id: PR:000026073

name:mitochondrial isocitrate dehydrogenase complex (NAD+) B (mouse)

def: A mitochondrial isocitrate dehydrogenase complex using NAD+ whose components are encoded in the genome of mouse containing isoform 2 of subunit alpha" [PRO:hjd]

is_a:PR: 000026071! mitochondrial isocitrate dehydrogenase complex (NAD+) (mouse)

relationship: has_part PR:000025356 ! isocitrate dehydrogenase [NAD] subunit alpha, mitochondrial isoform 2 (mouse)

relationship: has_part PR:000025359 ! isocitrate dehydrogenase [NAD] subunit beta, mitochondrial (mouse)

relationship: has_part PR:000025360 ! isocitrate dehydrogenase [NAD] subunit gamma, mitochondrial (mouse)

relationship: only_in_taxon taxon:10090! Mus musculus

#### Use Case 4. The Respiratory Chain IV Complex: Representing uncertainty in complex components

Cytochrome c oxidase, also known as Complex IV (EC 1.9.3.1), is a large transmembrane protein complex located in the inner mitochondrial membrane. Its function is sequestration of free radicals, coupled to the transport of protons across the inner mitochondrial membrane, which creates the proton gradient used by mitochondrial ATP synthase during ATP production. Although the function of Complex IV is well understood, the exact composition of the complex is not certain due to apparent genetic redundancy. Complex IV is composed of 13 polypeptides: three encoded in the mitochondrial genome, and ten encoded in the nuclear genome [22]. The uncertainty for this complex stems from the fact that some components of the complex are known (or predicted) to be encoded by multiple genes in the nuclear genome. The PRO stanza for Complex IV shown below illustrates how *has_part *statements that refer to "either-or" classes of proteins are used to represent uncertainty in a compact form with no loss of information. The *union_of *statement (highlighted by bold text in the stanza below) indicates which genes could code for a particular protein component. The *union_of *tag appears in the appropriate protein component stanzas, not in the protein complex stanza. These tags are shown below (as comments) for illustration purposes only.

[Term]

id: PR:000026295

name: respiratory chain complex IV (mouse)

def: A mitochondrial respiratory chain complex IV whose components are encoded in the genome of mouse. [PRO:CJB, PMID:8638158, PMID:21211513]

is_a: GO:0005751 ! mitochondrial respiratory chain complex IV

relationship: has_part PR:000025364 {cardinality = "1"} ! cytochrome oxidase subunit 1, mitochondrial (mouse)

relationship: has_part PR:000025366 {cardinality = "1"} ! cytochrome oxidase subunit 2, mitochondrial (mouse)

relationship: has_part PR:000025367 {cardinality = "1"} ! cytochrome oxidase subunit 3, mitochondrial (mouse)

relationship: has_part PR:000026286 complex IV component 4 (mouse)

! union_of: PR:000025368 ! Cox4i1

! union_of: PR:000025369 ! Cox4i2

relationship: has_part PR:000026294 {cardinality = "1"} ! cytochrome c oxidase subunit 5A, mitochondrial, transit peptide removed form (mouse)

relationship: has_part PR:000025371 {cardinality = "1"} ! cytochrome c oxidase subunit 5B (mouse)

relationship: has_part PR:000026287 {cardinality = "1"} ! complex IV component 6a (mouse)

! union_of: PR:000025372 ! Cox6a1

! union_of: PR:000025373 ! Cox6a2

relationship: has_part PR:000026288 {cardinality = "1"} ! complex IV component 6b (mouse)

! union_of: PR:000025374 ! Cox6b1

! union_of: PR:000025375 ! Cox6b2

relationship: has_part PR:000025376 {cardinality = "1"} ! cytochrome c oxidase subunit 6C (mouse)

relationship: has_part PR:000026289 {cardinality = "1"} ! complex IV component 7A (mouse)

! union_of: PR:000025377 ! Cox7a1

! union_of: PR:000025378 ! Cox7a2

! union_of: PR:000025379 ! Cox7a2l

relationship: has_part PR:000027496 {cardinality = "1"} ! complex IV component 7B (mouse)

! union of PR:000027491 ! Cox7b

! union of PR:000027489 ! Cox7b2

relationship: has_part PR:000025383 {cardinality = "1"} ! cytochrome c oxidase subunit 7C, mitochondrial (mouse)

relationship: has_part PR:000026290 {cardinality = "1"} ! complex IV component 8 (mouse)

! union_of: PR:000025380 ! Cox8a

! union_of: PR:000025381 ! Cox8b

! union_of: PR:000025382 ! Cox8c

relationship: only_in_taxon taxon:10090 ! Mus musculus

While not illustrated in the use cases above, many PRO complexes have species-specific protein types of modified isoforms. A few examples are: (i) PR:000025933 smad2-smad4 protein complex 1 (human), which contains active phosphorylated form of smad2 (MAD homolog 2), (ii) PR:000026035 myc-max acetylated complex (human), where both myc (myelocytomatosis oncogene) and max (Max protein) are acetylated, and (iii) PR:000027084 IRF3-P:IRF7-P complex (human), which contains the active phosphorylated forms of IRF3 (interferon regulatory factor 3) and IRF7 (interferon regulatory factor 7).

#### Viewing Protein Complex Data in PRO

**Figure 3:**
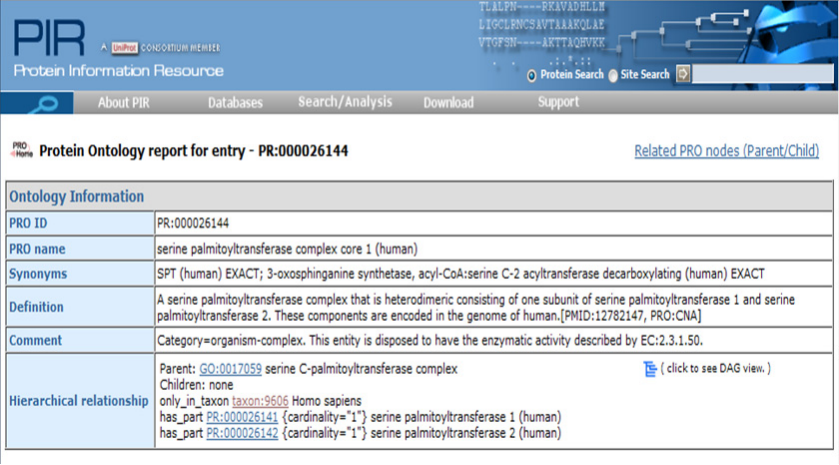
**Screenshot of the PRO entry for human serine palmitoyltransferase complex core 1 (SPT) as displayed on the PRO web site**.

The web display of the ProComp stanza for the human SPT complex described above in Use Case 2 http://purl.obolibrary.org/obo/PR_000026144 is shown in Figure 3. The web page provides human-readable access to entries in ProComp as well as navigation to detailed records about the complex subunits and related nodes in PRO.

#### Annotating Protein Complexes

The ProComp ontology provides species-specific, accessioned protein complex entities that support robust, context-specific annotation of protein biology [23]. Comprehensive functional annotation of proteins and protein complexes is not a mission of the PRO consortium; however, by providing uniquely accessioned protein complex entities PRO provides a critical and necessary resource to support unambiguous sharing of annotations by specialists across multiple biological disciplines. To support data exchange and integration by model organism and pathway database groups that are annotating protein complexes, biological annotations of proteins and protein complexes can be represented in Protein Annotation Files (PAF). PAFs are modeled on the Gene Annotation Files (GAFs) generated for GO annotations http://www.geneontology.org/GO.format.gaf-1_0.shtml. The PAF format consists of a standard header and 20 tab-delimited columns (11 required; 9 optional; see ftp://ftp.pir.georgetown.edu/databases/ontology/pro_obo/PAF_guidelines.pdf).

**Figure 4:**
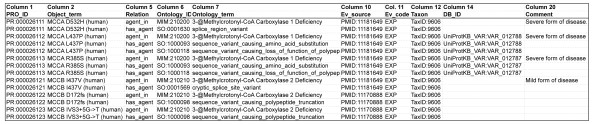
**Example of a Protein Annotation File (PAF) showing biological annotations for MCCC1 (MCCA) and MCCC2 (MCCB) human variants**. Only columns with information are shown and short names of the variants are used. This PAF file is available via the PRO ftp site: ftp://ftp.pir.georgetown.edu/databases/ontology/pro_obo/.

Figure 4 illustrates how a PAF is used to provide biological context for proteins and protein complexes for the MCC complex described in Use Case 1 above. 3-Methylcrotonylglycinuria is an inborn error of leucine catabolism caused by deficiency of 3-methylcrotonyl-CoA carboxylase activity (MCC; EC 6.4.1.4). Knowledge that mutations in human genes that encode the protein components of the MCC complex: MCCC1 (subunit alpha) and MCCC2 (subunit beta), cause methylcrotonylglycinuria type I (OMIM #210200) and methylcrotonylglycinuria type II (OMIM #210210), respectively, are represented in the PAF file (ftp://ftp.pir.georgetown.edu/databases/ontology/pro_obo/, and Figure 4). As shown, each variant of MCCC1 and MCCC2 has its own PRO ID and annotation. Annotations of variants may include sequence-related attributes (annotated with Sequence Ontology [24] terms; SO), relation to disease (annotated with MIM [25]), and/or functional information (annotated with GO). In this particular case the SO annotation provides important information regarding the sequence nature of the variant. Two of the variants in Figure 4 are generated because of missense mutations that, while within exons, alter RNA splicing either by removing a splice site (MCCAD532H, PR:000026111), or by activating a cryptic splice donor site (MCCB I437V, PR:000026121) [26]. In addition, the PAF makes it possible to indicate sequence variants which alter a biological process or function, as in MCCA L437P.

## Conclusions

The uniquely identified, species-specific protein complex classes in ProComp serve as a framework for formal representation of these biological entities and their biological contexts. For example, Na+-K+-ATPases, or "sodium pumps" create both an electrical and chemical gradient across the plasma membrane. Sodium pumps are usually tetramers consisting of two catalytic alpha, and two membrane-bound beta subunits. However, in high osmolarity environments such as kidney epithelium, a third (gamma) subunit is required for their proper functioning [27,28]. In mammals, each of the subunits is encoded by members of a multi-gene family and different subunit combinations likely provide tissue and developmental specificities of the entities exercising sodium pump functions. As illustrated in this manuscript, the design of the ProComp ontology supports the representation of the compositional complexity of protein complexes and, combined with information from manually-curated databases such as Reactome and MouseCyc, facilitates robust annotation of rich biological contexts as well. Future directions for data acquisition and curation within the ProComp project include working with a wide range of community bio-curation resources and investigators to create protein complex records that support curation of experimental annotations. We are developing software to convert PRO-relevant information from database resources into an ontological format. We are also extending an existing web-based data entry tool http://pir.georgetown.edu/cgi-bin/pro/race_pro that will support direct submission of protein complexes to PRO. The representation of protein complexes classes in PRO will be revisited frequently as additional information and evidence about their composition and stochiometry is identified in the scientific literature by PRO curators.

Improvements in the PRO ontology and annotation have come to light by our work on the use cases described in this manuscript. We outline a number of known issues that serve as the basis for future work. First, while the assertions in stanzas within PRO are intended to be backed by evidence, the distinction between experimental and inferential evidence (both legitimate) is not yet captured clearly. This is especially true in cases where the evidence for composition and stochiometry for protein complexes is derived from orthologous proteins and complexes in other organisms. Usefully recording such information, as we recognize is important, was hampered by deficits in our curation tools that made it impossible to record evidence for individual relations or, more specifically, cardinality. We are working with the Ontology for Biomedical Investigations (OBI; http://obi-ontology.org/) group on the development and application of an evidence code ontology to describe the evidence used to support protein complex assertions in ProComp, and with developers of the curation tools to enable capturing such evidence with appropriate granularity. Second, our current work records only protein monomer components of complexes facilitated by an interface that makes selection of the monomers and their identifiers manageable. However, as we note, protein complexes (such as Complex IV in Use Case 4) are known to have lipid, heme, and other co-factors as components. Extensions to our curation tools will be developed in order to select and include such components, identifiers for which are provided by, e.g., the Chemical Entities of Biological Interest (CheBI) ontology [29]. Third, there is further work to be done on the relations that connect protein complex and gene product mutation variants to disease. We have used *has_agent *and *is_agent_in *relations in our annotations to connect, provisionally, proteins with representation of sequence variation from the Sequence Ontology. However this does not agree with the formal definition in which these relations connect processes with their participants [30]. We plan to work with developers of the Relation Ontology to define appropriate relations. Fourth, we will work to improve the axioms we use to better enable use of reasoners to check consistency and facilitate advanced query. For example, ProComp currently uses *has_part *to relate a protein complex to its components and uses cardinality restriction to represent stoichiometry. However, because *has_part *is a transitive relation, the ontology falls outside OWL2-DL making it not possible to use standard reasoners. Thus, we are evaluating the possibility of replacing *has_part *with a new relation that is a non-transitive sub-property of the *has_part *relation. Finally, we will implement closure axioms that would more clearly define the number and kind of complex components. For example, our representation of Complex IV allows that there might be additional protein components because of the open world assumption. A closure axiom would assert that exactly 13 of the components of this complex are proteins, while still allowing that there might be other kinds of components.

## Availability and requirements

The Protein Ontology resource can be accessed on-line http://pir.georgetown.edu/pro/. Users can search PRO using accession IDs (e.g. PRO, UniProtKB, GO) or text (e.g., term name, definitions, synonyms). Searches can be restricted to specific modified forms (phosphorylated, etc.), database, or membership in a complex. PRO entries can also be accessed via hypertext links on gene detail pages in the Mouse Genome Informatics (MGI) database and from MouseCyc, Reactome, and EcoCyc. PRO (Release 20) contains 168 complexes drawn from Reactome (human), MouseCyc (mouse), EcoCyc (*E. coli*) and direct submissions from collaborating research groups. OBO Edit, an open source ontology editing tool http://oboedit.org/[31], can be used to view all of the relationships represented in protein complex stanzas from ProComp.

Existing conversion tools allow for OBO-formatted ontologies to be converted into Web ontology language (OWL) [32,33]. The PRO ontology is available in a number of formats, including OWL (latest version always at http://purl.obolibrary.org/obo/pr.owl).

## Competing interests

The authors declare that they have no competing interests.

## Authors' contributions

CJB, HD, AE, AR and PD researched and created protein complex stanzas for mouse and human. DN CA, and AR validated the ontology stanza formats and created the appropriate records in the PRO database. NR built software infrastructure to facilitate semi-automated acquisition of protein complex data from contributing databases. All authors contributed to the development of the formalisms to represent the classes of protein complexes illustrated in this manuscript. All authors participated in the writing of the manuscript. All authors read and approved the manuscript.
